# The enrichment of an alkaliphilic biofilm consortia capable of the anaerobic degradation of isosaccharinic acid from cellulosic materials incubated within an anthropogenic, hyperalkaline environment

**DOI:** 10.1093/femsec/fiv085

**Published:** 2015-07-20

**Authors:** C. J. Charles, S. P. Rout, E. J. Garratt, K. Patel, A. P. Laws, P. N. Humphreys

**Affiliations:** 1 Department of Biological Sciences, School of Applied Sciences, University of Huddersfield, Queensgate, Huddersfield HD1 3DH, UK; 2 Department of Chemical Sciences, School of Applied Sciences, University of Huddersfield, Queensgate, Huddersfield HD1 3DH, UK

**Keywords:** ISA, Isosaccharinic acid, Biofilm, EPS, hyperalkaline

## Abstract

Anthropogenic hyperalkaline sites provide an environment that is analogous to proposed cementitious geological disposal facilities (GDF) for radioactive waste. Under anoxic, alkaline conditions cellulosic wastes will hydrolyze to a range of cellulose degradation products (CDP) dominated by isosaccharinic acids (ISA). In order to investigate the potential for microbial activity in a cementitious GDF, cellulose samples were incubated in the alkaline (∼pH 12), anaerobic zone of a lime kiln waste site. Following retrieval, these samples had undergone partial alkaline hydrolysis and were colonized by a Clostridia-dominated biofilm community, where hydrogenotrophic, alkaliphilic methanogens were also present. When these samples were used to establish an alkaline CDP fed microcosm, the community shifted away from Clostridia, methanogens became undetectable and a flocculate community dominated by *Alishewanella* sp. established. These flocs were composed of bacteria embedded in polysaccharides and proteins stabilized by extracellular DNA. This community was able to degrade all forms of ISA with >60% of the carbon flow being channelled into extracellular polymeric substance (EPS) production. This study demonstrated that alkaliphilic microbial communities can degrade the CDP associated with some radioactive waste disposal concepts at pH 11. These communities divert significant amounts of degradable carbon to EPS formation, suggesting that EPS has a central role in the protection of these communities from hyperalkaline conditions.

## Abstract (precis)

A biofilm forming community able to degrade ISA was isolated from a hyperalkaline contaminated site.

## Graphical abstract

**Graphical Abstract Figure.**
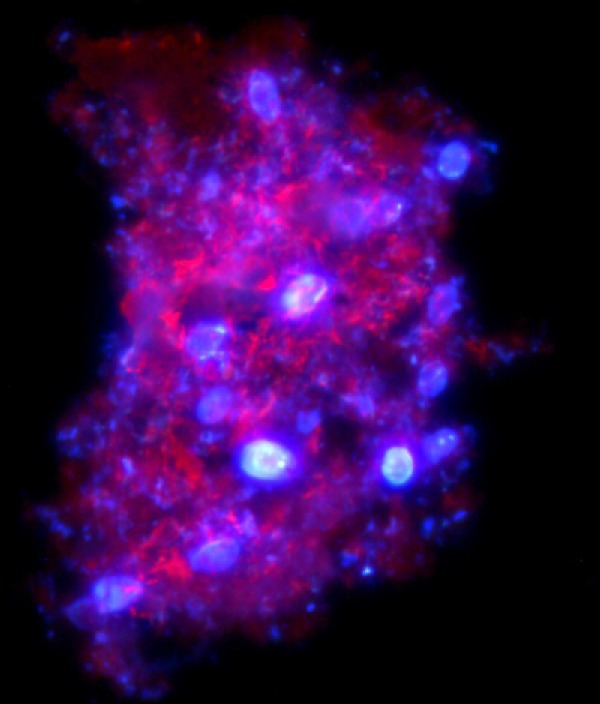
A biofilm forming community able to degrade ISA was isolated from a hyperalkaline contaminated site.

## INTRODUCTION

The UK's national nuclear waste legacy contains approximately 290 000 m^3^ (N.D.A. 2013) of intermediate-level radioactive wastes (ILW) which includes an estimated (∼2000 tonnes) (N.D.A. 2010a) of cellulosic materials (wood, paper and cloth) (Humphreys, Laws and Dawson 2010). One of the proposed strategies for the disposal of this ILW is a deep geological disposal facility (GDF) (N.D.A. 2010a) employing a multibarrier system which is likely to include a cement-based backfill (Chapman and Hooper 2012). Upon the closure of such a facility, groundwater ingress combined with corrosion processes will result in the development of a chemically reducing high pH (pH 12.5) environment (N.D.A. 2010b; Libert *et al.* 2011). Under these conditions, the cellulose portion of ILW is expected to undergo chemical, alkaline hydrolysis to form a variety of cellulose degradation products (CDP) (Knill and Kennedy 2003; Humphreys, Laws and Dawson 2010).

CDP is comprised of the alpha and beta diastereomers of isosaccharinic acid (ISA), alongside other small chain organic compounds including acetic acid (Van Loon and Glaus 1997; Motellier, Richet and Merel 1998; Knill and Kennedy 2003). The diastereomers of ISA are of significance when considering the performance of a GDF as they possess the ability to enhance the mobility of a range of radionuclides, including nickel, thorium, plutonium and uranium through complexation (Greenfield *et al.* 1992; Warwick *et al.* 2003; Allard and Ekberg 2006). In addition, the hemicellulose fraction of cellulosic waste components will also undergo anoxic, alkaline hydrolysis to form an additional 5-carbon form of ISA, known as xyloisosaccharinic acid (X-ISA) (Almond *et al.* 2012). Recent work by Randall *et al.* (2013) suggests that X-ISA does not have the same complexation properties as the alpha and beta forms of ISA but could, however, represent a source of organic carbon available for microbial metabolism.

Although the harsh geochemical conditions of an ILW-GDF place limitations upon microbial life, it may not prevent microbes from colonizing a facility. An investigation of an anthropogenic analogue of an ILW-GDF at a hyperalkaline contaminated site in Buxton, UK, where ISA is generated *in situ* (Rout *et al.* 2015) has revealed a microbially active site despite porewaters of up to pH 13 (Burke *et al.* 2012). The range of microbes present within the background sediments is diverse, with organisms within the Phyla Bacteroidetes, Proteobacteria and Firmicutes consistently making up large proportions of the sediment taxonomic profiles (Burke *et al.* 2012; Williamson *et al.* 2013; Bassil, Bryan and Lloyd 2014). The subsequent culturing of these sediments has shown that these communities are able to utilize the alpha form of ISA as a substrate under aerobic, nitrate, iron-reducing (Bassil, Bryan and Lloyd 2014) and methanogenic conditions (Rout *et al.* 2015). Sulphate reduction appears to be inhibited at pH > 10 (Bassil, Bryan and Lloyd 2014); however, the utilization of ISA under sulphate-reducing conditions has been observed at neutral pH indicating that this limitation is thermodynamic (Rizoulis *et al.* 2012; Rout *et al.* 2014). The heterogeneity of ILW and its compaction in grout may limit the availability of higher energy terminal electron acceptors such as nitrate and ferric iron, with the inundating ground water also depleted in these electron acceptors due to its passage through the microbial thermodynamic ladder (Bethke *et al.* 2011). Fermentation processes and subsequent methanogenesis therefore represent the most likely conditions to dominate an ILW-GDF

Microbes in nature can be found in biofilms of mixed syntrophic communities, with microbial biofilms found in a diverse range of environments (Summons *et al.* 2015; Urbieta *et al.* 2015). The secretion of extracellular polymeric substance (EPS) such as polysaccharides, proteins, lipids and nucleic acids during biofilm formation assists in bacterial survival and propagation (Flemming and Wingender 2010) and confers an increased resistance to environmental stresses such as pH and temperature fluctuations, desiccation and UV radiation (Jones *et al.* 1994; Gorlenko *et al.* 2004; Rodrigues *et al.* 2006; Ordoñez *et al.* 2009; Conrad *et al.* 2014). When considering the colonization of an ILW-GDF, the ability of microbes to migrate and adhere to niche areas such as ungrouted surfaces may allow for both microbial survival and growth under extreme alkaline conditions (Humphreys, West and Metcalfe 2010). The aim of this work was to culture, *in situ*, a biofilm forming consortium capable of colonizing cellulosic materials under anoxic, hyperalkaline conditions and to determine its ability to degrade CDP, which represent the primary organic carbon source within an ILW-GDF.

## METHODS

### Cellulose cotton preparation

In order to prepare the cellulose cotton for incubation, raw woven cotton fabric (Greige) was treated with NaOH to saponify the natural waxes along with an alkali stable phosphate ester detergent to emulsify the suspended impurities. Further treatment with NaOH and phosphonate stabilized H_2_O_2_ was carried out to bleach the fabric. The cotton was then rinsed, neutralized under acetic acid before finally being rinsed, dried and autoclaved at 121°C prior to use.

### Analogue site investigation

**Figure 1.**
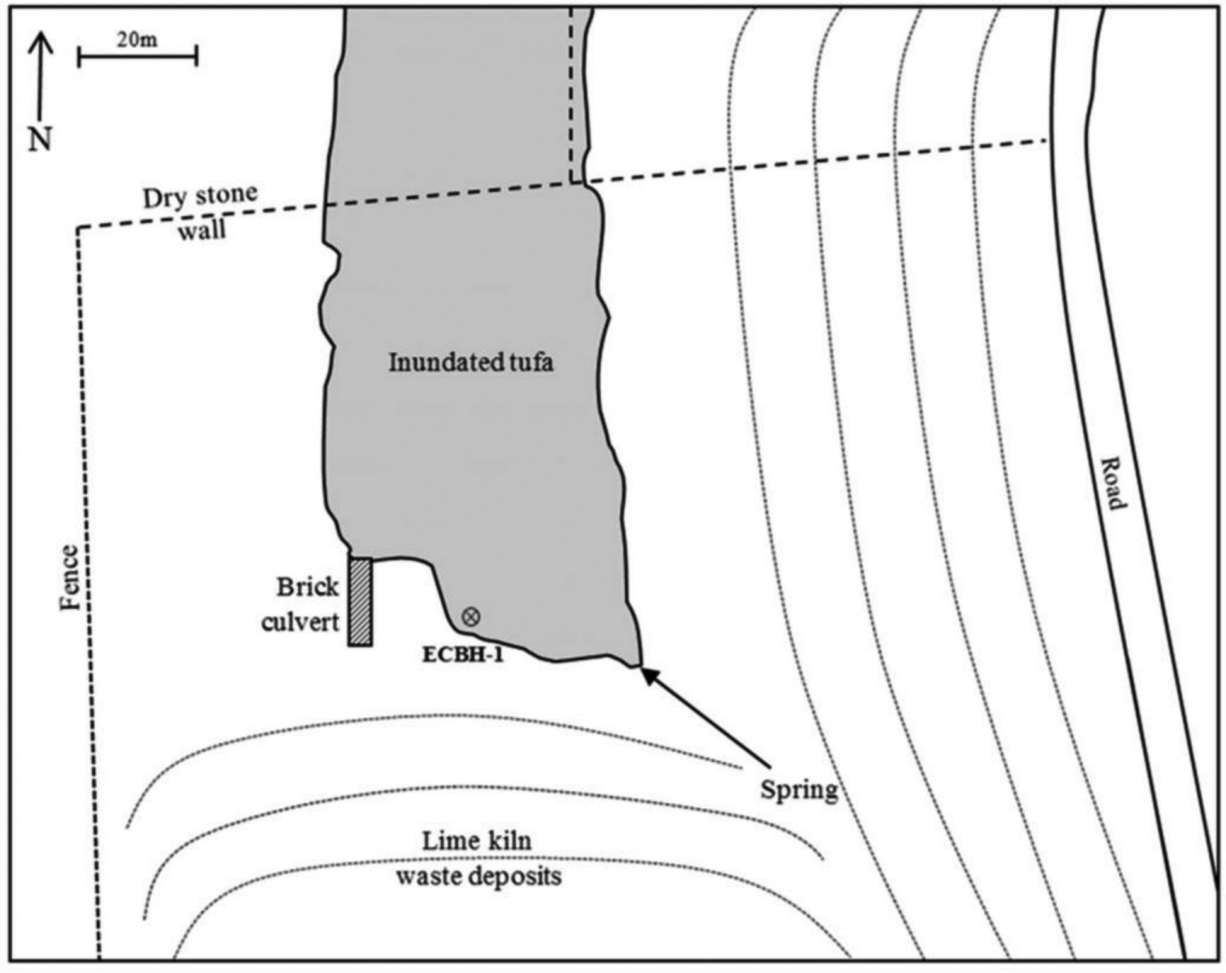
Overview of hyperalkaline contaminated site and position of emplaced cotton within bore hole 1 (ECBH-1).

During May 2014, a 2.2 cm Ø borehole was hand drilled to an approximate depth of 0.5 m into an area inundated with alkaline leachate at Brook Bottom, Harpur Hill, Buxton, UK (Fig. 1). An inert plastic liner with a perforated lower section was placed into the borehole. Approximately 5 g of sterile-treated cellulose cotton was loaded into a nylon mesh bag and placed at the bottom of the borehole. After a period of 3 months, the cotton was recovered along with sediment and porewater samples from the immediate vicinity of the sample. *In situ* pH and Eh values were determined prior to sample recovery using a handheld portable pH meter with calibrated electrodes and an InLab Redox Micro probe (Mettler Toledo, UK) tested in accordance with BS ISO 11271:2002 (B.S.I. 2002). All recovered materials were sealed in airtight containers along with anaerobic gas packs (Anaerogen, Oxoid, UK) for transport. Sediment and porewater samples were stored at −20°C until analysis and cotton not used for immediate studies was stored at −20°C in a solution of 140 mL of ultrapure water, 10 mL of 1M TRIS-HCl (pH7.5) and 250 mL of 96% ethanol after an overnight fixation step in 4% paraformaldehyde in phosphate-buffered saline (PBS).

Porewater, cotton and sediment ISA content was determined as previously described by Rout *et al.* (2014, 2015) against ISA standards in the alpha, beta and xylo conformations (Almond *et al.* 2012; Shaw *et al.* 2012). C1–8 volatile fatty acid (VFA) content of both the sediment and cotton was determined using a standard extraction method outlined in Eaton *et al.* (2005) and analysed via GC-FID as described by Rout *et al.* (2014).

### Microscopy

Scanning electron microscopy was undertaken using a JEOL JSM-6060LV microscope (JEOL, USA). Samples were dehydrated using a serial ethanol dilution of 25, 50, 75 and 100% for 2 min per step and then sputter coated via a gold palladium plasma (CA7625 Polaron, Quorum Technologies Ltd, UK). Fluorescence microscopy was carried out using an Olympus BX41 laboratory microscope (Olympus, USA). Live dead staining was carried out using the BAC light Live/dead kit (Life technologies, UK), fluorescein isothiocyanate (FITC) (Sigma-Aldrich, UK) staining was used for protein and visualization of individual bacteria cells and the polysaccharide components were achieved using ethidium bromide and Calcofluor White (Sigma-Aldrich, UK) staining, respectively. For DNase digestion, microcosm fluid (1 mL) was centrifuged at 10 000 × *g* for 1 min and resuspended in ultrapure water (1 mL). A 10-fold dilution of this was then subjected to digestion by DNase using a DNase 1 kit (Sigma-Aldrich, UK).

### Microcosm

In order to investigate ISA degradation, approximately 1 g of colonized cotton was washed with 10 mL N_2_ purged sterile PBS under an inert environment to remove any transient microorganisms. The washed cotton was then added to a continuously stirred microcosm containing 175 mL of pre-reduced 10% CDP and 90% mineral media (B.S.I. 2005) at pH 11 and 20°C that had been purged with nitrogen and maintained with a nitrogen headspace to ensure anoxic conditions. CDP was produced as previously described by Rout *et al.* (2014). The microcosm was brought up to a final volume of 250 mL by feeding 25 mL of CDP every 2 weeks with the pH adjusted using 4M NaOH every 7 days. After this period, the cotton was removed and the microcosm was switched to a 10% waste/feed cycle with CDP every 2 weeks. The microcosm was maintained with a nitrogen atmosphere and all reagents were reduced prior to use with disodium sulphide nonahydrate (Sigma-Aldrich, UK) and sodium dithionite (Fisher, UK) as per BS ISO 14853:2005 (B.S.I. 2005) and stored under nitrogen. Resazurin redox indicator (Fisher, UK) present within the mineral media provided an indication of anaerobic conditions within the microcosm and all manipulations of the microcosm were carried out under a stream of nitrogen to maintain anoxic conditions. Sufficient time (50 weeks) was allowed for the microcosm chemistry to stabilize and also to allow for the washout of any transient microorganisms. The microcosm was sampled every 2 days over 2 feed/waste cycles to determine the ISA and VFA content. For each sample period, microcosm fluid (1 mL) was taken, centrifuged at 10 000 × *g* for 1 min and the supernatant filter sterilized using a 0.45-μm syringe filter (Sartorius, UK) and stored at −20°C prior to analysis. The gas headspace (75 mL starting volume) was sampled every 2 days with the composition determined via gas chromatography using Agilent 6850 gas chromatograph (Hewlett Packard, UK) fitted with a HP-Plot/Q+ PT column and thermal conductivity detection (TCD). Headspace gas (100 μL) was removed using a lockable gas syringe from the microcosm and passed through the column under the following conditions: initial temperature of 60°C for 2 min, followed by an increase to 120°C at a ramp rate of 30°C min^−1^ with a detector temperature of 250°C. Gas headspace pressure was measured using a digital manometer (TPI, UK) before gas sample periods.

Microcosm fluid (1 mL) containing the suspended flocs was taken on days 0, 7 and 14 and spun at 10 000 × *g* for 1 min for ATP/biomass detection using a 3M Clean-Trace Biomass Detection Kit and Luminometer employing a modified method (3M, UK). The pellet was washed once with pH 4 PBS and reconstituted in pH 7 PBS to remove interference from excess alkalinity and salts. Following analysis, CFU mL^−1^ and dry weight biomass (DW) were calculated against a standard curve of *Escherichia coli* K12 concentrations. In addition, a set of control microcosms amended with 50 μg mL^−1^ chloramphenicol were prepared and were sampled as per the test microcosms. The controls served as an abiotic comparison for the elimination of sorption and precipitation events. All data were processed in Microsoft Excel with calculated means and associated standard error shown in all relevant results. Carbon flow calculations were undertaken using balanced equations 1 and 2 for the fermentation of ISA to acetate and hydrogen.

(1) \begin{equation*} {\rm ISA} + 4{\rm H}_{\rm 2} {\rm O} \to 2{\rm CH}_{\rm 3} {\rm COO}^ - + 2{\rm HCO}_3^ - + 4{\rm H}_2 + 4{\rm H}^ + \end{equation*}

(2) \begin{equation*} {\rm XISA} + 3.33{\rm H}_{\rm 2} {\rm O} \to 1.67{\rm CH}_{\rm 3} {\rm COO}^ - + 1.67{\rm HCO}_3^ - + 3.33{\rm H}_2 + 3.33{\rm H}^ + \end{equation*}

### Preparation of 16S rDNA clone libraries

Total genomic DNA was extracted from the cotton and microcosm using a Powersoil DNA extraction kit (Mo-BIO, Carlsbad, USA) with the following modifications. For the cotton, approximately 0.25 g was washed with pH 7.0 PBS and loaded into a glass bead tube with 100 μL β-marcaptoethanol and the bead beating step extended to 1 h in order to overcome dampening effects introduced by the material. For genomic DNA extraction from the microcosm, 25 mL of fluid was centrifuged at 5000 × *g* for 15 min and the pellet resuspended in 25 mL of pH 4.0 PBS. The sample was then centrifuged again at 5000 × *g* for 15 minutes and resuspended in 2 mL of pH 7.0 PBS. An amount of 1 mL of the concentrated sample was transferred to a 1.5 mL tube and centrifuged again at 10 000 × *g* for 1 min, after which the supernatant was removed and the cell pellet resuspended in the reaction fluid provided in the glass bead tubes of the Powersoil kit. The resulting mixture was then transferred back to a glass bead tube and bead beaten with 100 μL β-marcaptoethanol for an increased time of 20 min to overcome clogging due to the EPS and then run as per the supplier's instruction. These modifications were found to increase the yield and purity of DNA obtained from both samples by removing excess salts, inhibiting nucleases and neutralizing the samples.

Purified genomic DNA was quantified and quality checked by spectroscopic methods and used as a template to amplify the 16S rRNA gene. A ∼1500 bp fragment of the Eubacterial 16S rRNA gene was amplified using broad specificity primers pA and pH (Edwards *et al.* 1989), and a ∼750 bp fragment of the archaeal 16S rRNA gene was amplified using primers Ar and Af (Gantner *et al.* 2011). PCR reactions were carried out using BIOMIX red master mix (BIOLINE, UK) with PCR fragments purified via a Qiaquick PCR purification kit (Qiagen, UK) and visualized using a 1.0% agarose TAE gel with SYBR® Safe staining (Life technologies, UK). PCR products were ligated into the standard cloning vector PGEM-T easy (Promega, USA) and transformed into *E. coli* JM109 competent cells (Promega, USA). Transformed cells were grown on Luria–Bertani (LB) agar containing 100 μg mL^−1^ ampicillin overlaid with 40 μL of 100 mM IPTG and 40 μL of 40 mg mL^−1^ X-GAL (5-bromo-4-chloro-3-indolyl-β-D-galactopyranoside) in N′N dimethylformamide for blue–white colour screening. Insert-containing colonies were transferred to 96 well plates containing LB agar with 150 mg mL^−1^ ampicillin and sequenced using Sanger sequencing technology (GATC Biotech, Germany). Inserts were amplified using a T7 forward primer and the resulting 16S rRNA gene sequences aligned using the multiple sequence alignment package MUSCLE (www.ebi.ac.uk/Tools/services/web/toolform.ebi?tool=muscle) and chimera checked using the UCHIME component of the Mothur suite, where chimeric sequences were omitted from the analysis (Schloss *et al.* 2009). Sequences were analysed against the NCBI database using Basic Local Alignment Search Tool (MegaBLAST) utilizing the 16S ribosomal RNA sequences for Bacteria and Archaea (Altschul *et al.* 1997). Phylogenetic families were then determined at a 95% confidence level by comparison with the Ribosomal Database Project (Cole *et al.* 2009).

### Nucleotide accession numbers

The 16S rRNA sequence data from the colonized cotton were submitted to GenBank under accession numbers KP263977–KP264111 and the microcosm sequences under the numbers KP728118–KP728176.

## RESULTS

### Chemical and physiological cotton analysis

The pH in the vicinity of the cotton samples was between 11.5 and 12.0 and redox measurements were found to be negative in both the associated sediment (−77 mV) and porewaters (−66 mV). Both the alpha and beta forms of ISA were extracted from the cotton (>0.5 mg (g dry wt)^−1^), the sediment (>0.5 mg (g dry wt)^−1^)) and porewater (7.64 mg L^−1^ alpha, 6.82 mg L^−1^ beta) (Table 1) indicating *in situ* alkaline cellulose hydrolysis (Knill and Kennedy 2003).

**Table 1.** Analysis of porewater, sediment and cotton retrieved from sample borehole.

Source	pH	eH	Acetate	α-ISA	β-ISA
Porewater (mg L^−1^)	11.92	−66.00	208.90	7.64	6.82
Sediment (mg (g dry wt)^−1^)	11.50	−77.00	127.24	1.01	0.54
Cotton (mg (g dry wt)^−1^)	N/S*	N/S	141.16	2.34	0.85

*N/S-Not sampled.

**Figure 2.**
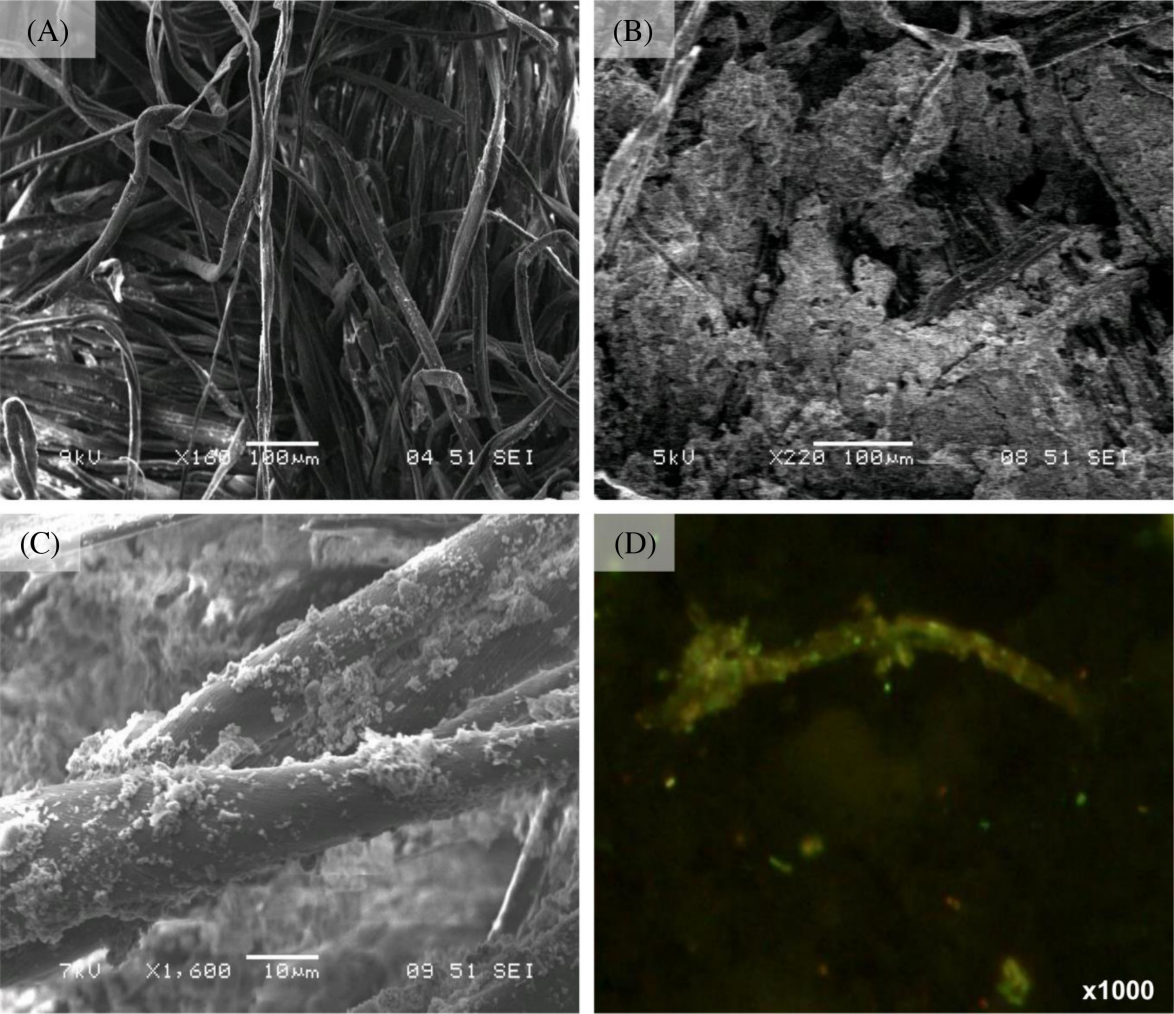
Microscopy investigation of the cellulose cotton. (**A**) Sterile cotton. (**B**) Cellulose cotton from the borehole showing biofilm formation. (**C**) Close up of individual fibre showing individual cells, EPS aggregates and mineral precipitate. (**D**) Live/dead image of individual cotton fibre.

The surfaces of the colonized cotton showed areas of EPS indicative of biofilm formation and surface-associated mineral precipitates (Fig. 2B and Fig. S1, Supporting Information) with individual viable bacterial cells being visible on some fibres (Fig. 2C and D).

### 16S rDNA profile of colonized cotton

**Figure 3.**
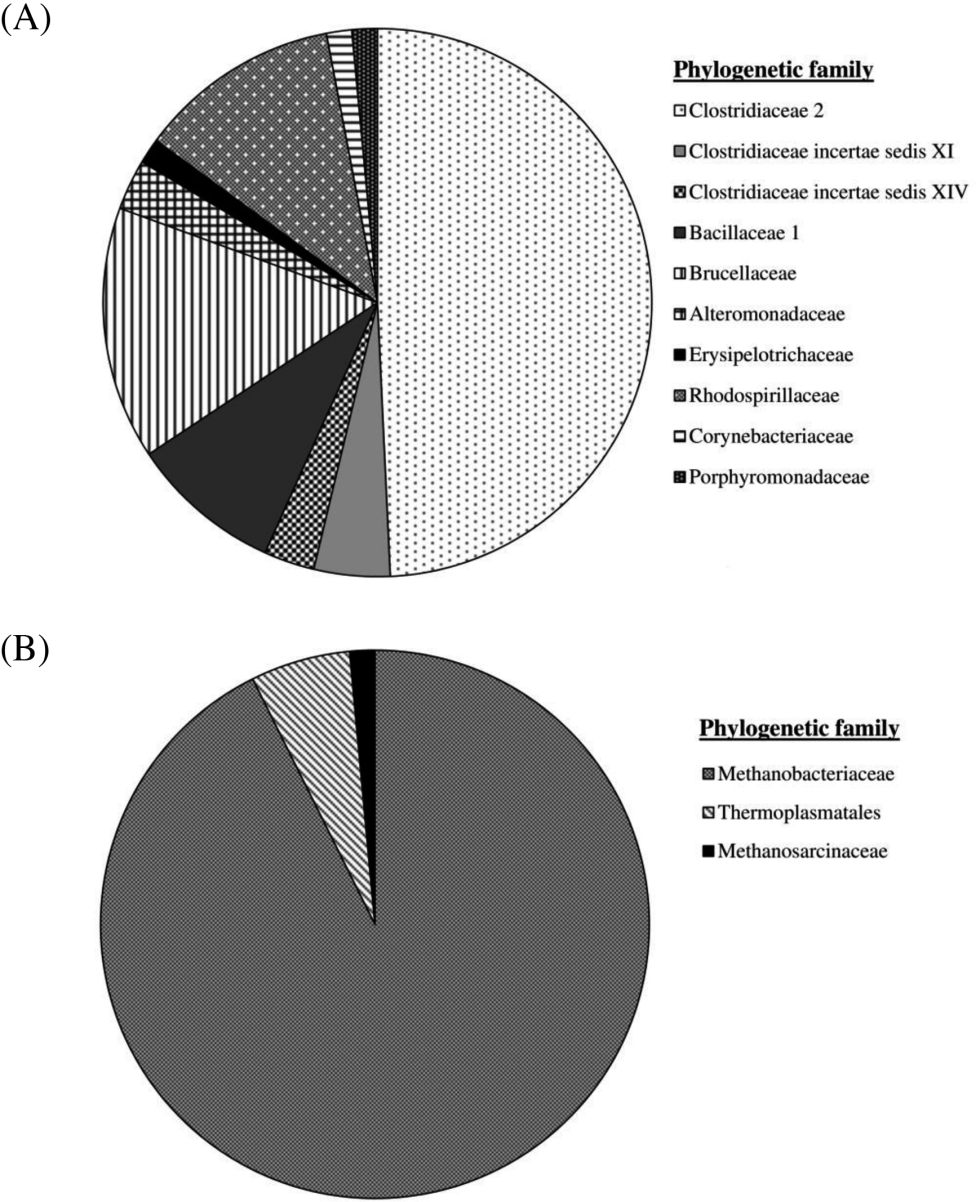
16S rRNA gene clone libraries of the colonised cotton. (**A**) Eubacterial (*n* = 67). (**B**) Archaeal (*n* = 68). Phylogenetic families were assigned to clones through a MegaBLAST database search.

The cotton's Eubacterial clone library (Table S1, Supporting Information) was dominated by the order Clostridiales which represented 58% of the clones obtained (*n* = 67, Fig. 3A). Of these Clostridia, 33 sequences most closely matched organisms from the family Clostridiaceae 2, where 13 sequences most closely matched *Clostridium formicaceticum* strain DSM 92 (95% sequence similarity) and a further 10 to *Anaerovirgula multivorans* strain SCA (97% sequence similarity). The remaining nine clones most closely matched sequences belonging to the genus *Alkaliphilus*, of which eight were closely related to *Alkaliphilus oremlandii* strain OhILAs (91–93% sequence similarity) and one related to *A. transvaalensis* strain SAGM1 (98% sequence similarity). The remaining clones of the Clostridia were represented by sequences most closely related to organisms from the families *Clostridium incertae sedis XI* (three sequences) and *Clostridium incertae sedis XIV* (two sequences).

The remainder of the clone library was made up of a diverse range of taxonomic families (Table S1, Supporting Information) including representatives from Brucellaceae, primarily related to *Ochrobactrum anthropi* strain ATCC 49188 (96–99% sequence similarity); Corynebacteriaceae, dominated by *Corynebacterium marinum* strain D7015 (98–99% sequence match similarity); and the Bacillaceae 1, dominated by *Bacillus pseudofirmus* strain OF4 (89–99% sequence similarity).

The Archaeal clone library (Table S2, Supporting Information) was dominated (93%) by sequences most closely matching *Methanobacterium alcaliphilum* strain NBRC 105226 (99% sequence similarity) (*n* = 68, Fig. 3B). The remaining sequences were most closely related to *Methanomassiliicoccus luminyensis* strain B10 (4 sequences 89% sequence match) and *Methanosarcina mazei* Go1 (99% sequence similarity).

### CDP-driven microcosms

**Figure 4.**
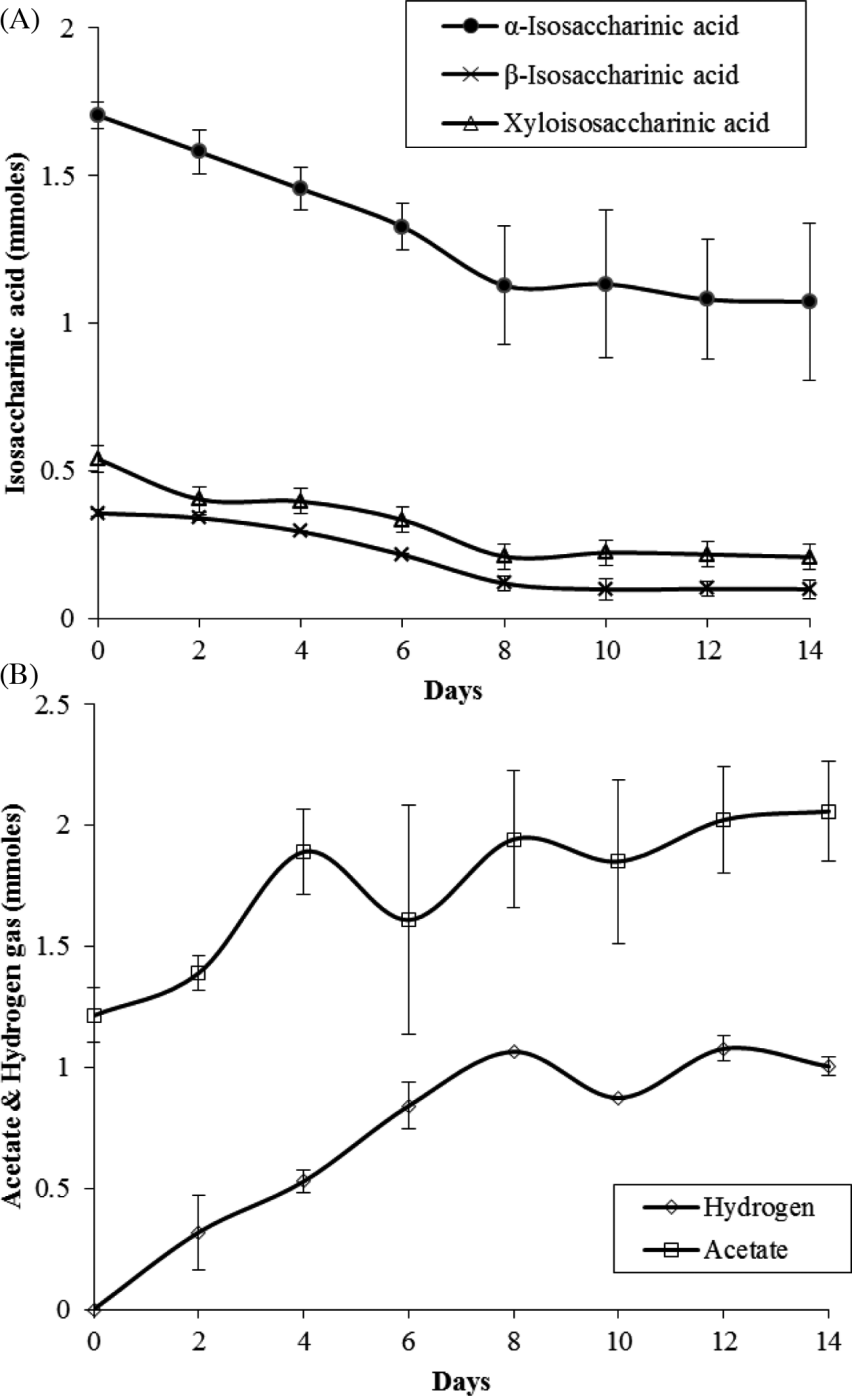
Chemistry of the CDP-driven pH 11 microcosm over two waste/feed cycles using colonized cellulose cotton as an inoculation source. (**A**) Alpha, beta and xylo isosaccharinic acid degradation profile. (**B**) Hydrogen and acetate production profile.

**Figure 5.**
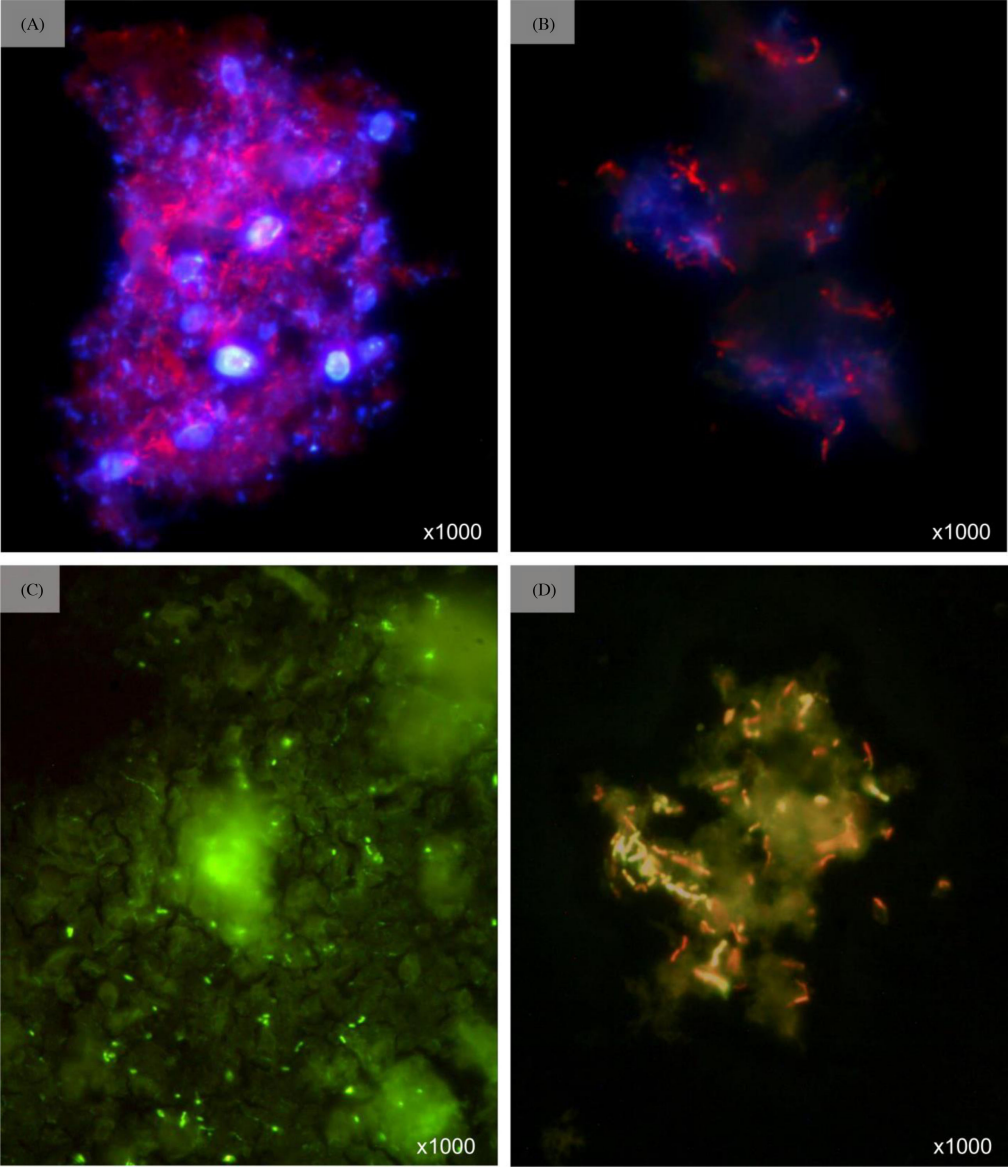
Microscopy investigation into the morphology of the pH 11 microcosm. (**A**) Ethidium bromide and Calcofluor white stain of bacterial flocculate showing individual cells and eDNA (red) and extracellular polysaccharides (blue). (**B**) DNase digest of flocculates stained with ethidium bromide and Calcofluor white. (**C**) FITC strain of bacterial flocculate showing areas containing protein (green). (**D**) Live/dead image of bacterial flocculate.

The microcosm demonstrated significant degradation of ISA at pH 11.0 over two waste/feed cycles (Fig. 4) with first-order rate constants of 3.33 × 10^−2^ day^−1^ (SE ± 2.0 × 10^−2^) for alpha, 9.36 × 10^−2^ day^−1^ (SD ± 2.2 × 10^−2^) for beta and 6.78 × 10^−2^ day^−1^ (SE ± 2.85 × 10^−2^) for X-ISA. Acetate was the only VFA detected and gradually accumulated in the system reaching a peak of 2.06 mmoles (SE ± 0.2), similarly hydrogen gas accumulated in the headspace over the course of the feed cycle reaching 1.00 mmoles (SE ± 0.04). Neither carbon dioxide nor methane was detected in the headspace of the microcosm; however, soluble inorganic carbon increased within the system (data not shown) with the pH after each cycle having an average pH of 10.8 (SE ± 0.4). The CDP fed microcosm inoculated with the colonized cotton was dominated by polymicrobial flocs with fluorescence microscopy showing microbial cells embedded in an EPS composed of protein, polysaccharide and extracellular DNA (eDNA) (Fig. 5).

Measurement of the ATP concentration of the microcosm showed that cell density increased over the feed/waste cycles (Table S4, Supporting Information) indicating that a portion of the organic carbon was used for the generation of both cell biomass and EPS. Carbon flow calculations (Rittmann and Mccarty 2001) based on the degradation of ISA showed 23.7% of the carbon was converted to acetate and 12.1% converted to carbonate from energy generating processes, 0.5% was converted to cell biomass and a further 63.7% was theorized to be involved in processes relating to EPS production. The yield of dry cell biomass was 0.012 mg (mg ISA)^−1^ degraded, the system could not be stoichiometrically balanced due to the unknown composition of the flocculate EPS material. Comparison of the samples amended with chloramphenicol showed no ISA degradation and the production of acetate and hydrogen was not detected (Fig. S2, Supporting Information) indicating that ISA degradation was via microbial activity rather than chemical processes or sorption.

### Microcosm clone library

**Figure 6.**
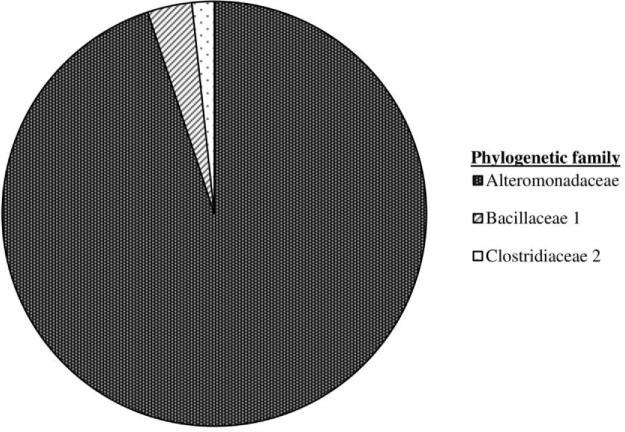
Eubacterial (*n* = 59) 16s rRNA gene clone library of pH 11 CDP-driven microcosm.

The microcosm microbial populations demonstrated a significant shift away from that associated with the emplaced cotton samples, with Archaeal taxa no longer being detectable and the Eubacterial population no longer dominated by the Clostridiales. The environmental and physiological constraints imposed within the microcosm resulted in a population dominated by clones of *Alishewanella jeotgali* strain MS1 (99% sequence similarity) from the family Alteromonadaceae (Table S3, Supporting Information; Fig. 6). The remaining clones included representatives of the family Bacillaceae, most closely matching *B. pseudofirmus* strain OF4 (98% sequence similarity) and *A. crotonatoxidans* strain B11–2 (98% sequence similarity) of the family Clostridiaceae 2.

## DISCUSSION

Previous authors noted the presence of an organic electron donor within the soils at Harpur Hill that allowed for electron flow into nitrate and iron-reducing processes at depth (Burke *et al.* 2012). The generation of CDPs from the site's soil organic matter has been demonstrated (Rout *et al.* 2015) and in this study the addition of cotton cellulose resulted in its partial alkaline hydrolysis to CDPs with the concentration of alpha and beta ISA in the porewater and sediments being higher than those measured by Rout *et al.* (2015). This supports the concept that the hyperalkaline conditions created at this site are capable of generating CDP. The presence of acetate, a common end product of ISA fermentation (Bassil, Bryan and Lloyd 2014; Rout *et al.* 2014, 2015), in the porewater, sediment and cotton indicated an active anaerobic microbial community in the immediate proximity of the cotton even though the ambient pH was between pH 11.5 and 12.

Cotton fibres were covered with large areas of EPS indicative of biofilm formation (Fig. 2A and B) with individual cells being only rarely visible (Fig. 2C). This is a marked contrast to the colonization of cotton incubated in a landfill site under neutral anaerobic conditions reported by McDonald *et al.* (2012), where fibres were heavily colonized with cells and exhibited the characteristic pits and grooves associated with microbial cellulose hydrolysis. The reduced colonization of the cotton under the hyperalkaline conditions present at the site is further illustrated by the live/dead staining of the cotton (Fig. 2D) which revealed a low density of live cells on the individual cotton fibres and within the surrounding biofilm material. Previous work by Grant *et al.* (2002) demonstrated the ability of alkaliphilic microorganisms to form a biofilm upon the surface of the cementitious materials presumably to provide a degree of protection from the alkaline stresses imposed by the local environment. This formation of EPS as a response to hyperalkaline conditions is replicated in these microcosm studies where a polymicrobial, eDNA stabilized floc-based population developed (Fig. 5A). The importance of EPS generation in this system is illustrated by the fact that >60% of the available carbon is diverted to EPS formation, a finding similar to the carbon distribution in biofilm systems reported by Jahn and Nielsen (1998).

The microbial flocs were composed of an EPS containing protein, polysaccharides and eDNA. Polysaccharides are a common component of EPS and moderate a range of bacterial biofilm properties including adhesion, cell aggregation, cohesive nature, protection as well as the sorption of organic compounds and inorganic ions (Flemming and Wingender 2010). Imaging of the polysaccharide component revealed its distribution throughout the flocculate with large globular-like structures (Fig. 5A). DNAse treatment caused the loss of these structures resulting in a less compact structure of cells associated with polysaccharide, indicating a relationship between the eDNA and the distribution of the polysaccharide components (Fig. 5B). The role of eDNA within biofilms appears to serve a number of functions (Dominiak, Nielsen and Nielsen 2011), in this case it is likely to aid the structure and function of the flocculate community (Gloag *et al.* 2013). The presence of eDNA within the flocculate structure is also likely to act as a phosphate store for the constituent microbial consortia (Dell'anno and Danovaro 2005). Calcium ions are abundant at the site and as such the interaction between eDNA and these ions is likely to promote cell aggregation and biofilm formation within these alkaliphilic cultures (Das *et al.* 2014). This is illustrated by the fact that treatment of the flocs with DNase resulted in the loss of flocculate stability (Fig. 5A and B). Imaging of the protein component of the flocs showed large concentrated areas of protein within the flocculate (Fig. 5C). Protein serves a wide range of functions within biofilm including the permitting of redox activity, protection from environmental conditions, enzymatic reactions and sorption of organic compounds sorption and inorganic ions (Flemming and Wingender 2010).

The presence of the cotton cellulose within the sediments selected for organisms of the order Clostridia which contrasts with previous investigations of the background sediments where a larger degree of taxonomic diversity was observed (Williamson *et al.* 2013; Bassil, Bryan and Lloyd 2014), presumably due to greater diversity of energy sources and colonization from surrounding pasture land. Of the Clostridiaceae 2 species identified, *C. formicaceticum* has broad spectrum carbohydrate fermentation capabilities (Andreesen, Gottschalk and Schlegel 1970), but was not previously associated with alkaline conditions. This contrasts with species from the genera *Anaerovirgula and Alkaliphilus* which have all been previously associated with alkaline sites (Takai *et al.* 2001; Pikuta *et al.* 2006; Fisher *et al.* 2008).

The Archaeal population associated with the cotton was dominated by hydrogenotrophic, alkaliphilic *Methanobacterium* sp. showing sequence similarity to *M. alcaliphilum* (Worakit *et al.* 1986). These findings are in agreement with clone libraries generated from microcosms previously developed from sediment samples from the same site (Rout *et al.* 2015). Although these organisms are able to utilize acetate as a growth factor (Wu *et al.* 1992; Kotelnikova, Macario and Pedersen 1998), they are incapable of acetoclastic methanogenesis which accounts for the accumulation of acetic acid in extracts from the cotton and surrounding sediment and porewaters. In addition, a small number of sequences showing similarity to *Me. luminyensis* (Dridi *et al.* 2012) and *Methanosarcina* sp. (Maestrojuan *et al.* 1992) were also detected.

The microbial population established in the microcosm was much less diverse than that present on the cotton samples with the almost complete removal of Clostridia and the total loss of methanogens from the system. This resulting fermentative system was dominated (95% of clones) by organisms most closely related to *Alishewanella* sp., which was a minor component (3% of clones) of the population present on the colonized cotton. This facultative anaerobic genus is most commonly associated with fermented seafood, but has also been isolated from landfill soils (Kim *et al.* 2009, 2010; Jung, Chun and Park 2012; Kolekar *et al.* 2013). Its ability to grow in alkaline conditions up to pH 12 has also been reported (Kim *et al.* 2009, 2010; Tarhriz *et al.* 2012), and its ability to degrade a range of substrates appears to have enhanced its ability to thrive within the CDP-driven microcosm. The ability to form biofilms and pellicles has been reported in *A. jeotgali* which may indicate a pivotal role for the *Alishewanella* sp. in the formation and maintenance of the bacterial aggregates within the microcosm (Jung, Chun and Park 2012) (Fig. 5A).

A range of degradation rate constants for the various forms of ISA (alpha, beta and xylo) were observed in the derived microcosms. The rate constant of beta ISA degradation was similar to that reported by Rout *et al.* (2015) at pH 11, whilst the rate constant of alpha ISA degradation was greatly reduced, potentially due to the reduced role of key genera such as *Alkaliphilus* (Rout *et al.* 2015). This is the first time that a microbial degradation rate constant for xylo ISA has been published.

The loss of methanogens from the microcosm cannot be entirely attributed to the pH, since a pH 11.0 methanogenic microcosm has been successfully established using sediments from the Buxton site (Rout *et al.* 2015). In that case, a similar range of methanogens were observed to that identified here associated with the cotton but with a Eubacterial population dominated by *Alkaliphilus*. The lack of Clostridia species specifically *Alkaliphilus* sp. within the microcosm formed from the colonized cotton appears to have retarded the ability of the associated methanogenic population to become established. Tight adherence to the cotton fibres and a possible differences in redox potential between the internal biofilm environment and the enrichment media may have also contributed to the poor transition of the methanogens and Clostridia species leading to an *Alishewanella* dominated system (Sridhar and Eiteman 1999; Stuart *et al.* 1999).

The presence of cotton fibres with the hyperalkaline analogue site at Harpur Hill provided both a source of CDP to drive anoxic metabolism and a surface for microbial colonization. Subsequent subculturing indicated that the cotton provided a surface for the adherence of a narrow range of Clostridiaceae 2 species and promoted the development of a floc-based alkaliphilic population dominated by *Alishewanella* sp. able to degrade CDP up to a pH of 11.0. Although methanogenic populations were detected on the cotton fibres, they were unable to make the transition to floc-based suspended growth.

## Supplementary Material

Supplementary data are available at FEMSEC online

## References

[bib1] Allard S, Ekberg C (2006). Complexing properties of α-Isosaccharinate: stability constants, enthalpies and entropies of Th-complexation with uncertainty analysis. J Solution Chem.

[bib2] Almond M, Shaw PB, Humphreys PN (2012). Behaviour of xyloisosaccharinic acid and xyloisosaccharino-1, 4-lactone in aqueous solutions at varying pHs. Carbohyd Res.

[bib3] Altschul SF, Madden TL, Schäffer AA (1997). Gapped BLAST and PSI-BLAST: a new generation of protein database search programs. Nucleic Acids Res.

[bib4] Andreesen JR, Gottschalk G, Schlegel HG (1970). *Clostridium formicoaceticum* nov. spec. isolation, description and distinction from *C. aceticum* and *C. thermoaceticum*. Arch Microbiol.

[bib5] B.S.I. (2002). BS ISO 11271:2002 Soil Quality—Determination of Redox Potential— Field Method. S ISO 11271.

[bib6] B.S.I. (2005). BS ISO 14853:2005 Plastics-Determination of the Ultimate Anaerobic Biodegradation of Plastic Materials in an Aqueous System-Method by Measurement of Biogas Production. BS ISO 14853:2005.

[bib7] Bassil NM, Bryan N, Lloyd JR (2014). Microbial degradation of isosaccharinic acid at high pH. ISME J.

[bib8] Bethke CM, Sanford RA, Kirk MF (2011). The thermodynamic ladder in geomicrobiology. Am J Sci.

[bib9] Burke IT, Mortimer RJ, Palaniyandi S (2012). Biogeochemical reduction processes in a hyper-alkaline leachate affected soil profile. Geomicrobiol J.

[bib10] Chapman N, Hooper A (2012). The disposal of radioactive wastes underground. P Geologist Assoc.

[bib11] Cole JR, Wang Q, Cardenas E (2009). The Ribosomal Database Project: improved alignments and new tools for rRNA analysis. Nucleic Acids Res.

[bib12] Conrad R, Ji Y, Noll M (2014). Response of the methanogenic microbial communities in Amazonian oxbow lake sediments to desiccation stress. Environ Microbiol.

[bib13] Das T, Sehar S, Koop L (2014). Influence of calcium in extracellular DNA mediated bacterial aggregation and biofilm formation. PloS One.

[bib14] Dell'anno A, Danovaro R (2005). Extracellular DNA plays a key role in deep-sea ecosystem functioning. Science.

[bib15] Dominiak DM, Nielsen JL, Nielsen PH (2011). Extracellular DNA is abundant and important for microcolony strength in mixed microbial biofilms. Environ Microbiol.

[bib16] Dridi B, Fardeau M-L, Ollivier B (2012). *Methanomassiliicoccus luminyensis* gen. nov., sp. nov., a methanogenic archaeon isolated from human faeces. Int J Syst Evol Micr.

[bib17] Eaton AD, Clesceri LS, Rice EW (2005). Standard Methods for the Examination of Water and Wastewater.

[bib18] Edwards U, Rogall T, Blöcker H (1989). Isolation and direct complete nucleotide determination of entire genes. Characterization of a gene coding for 16S ribosomal RNA. Nucleic Acids Res.

[bib19] Fisher E, Dawson AM, Polshyna G (2008). Transformation of inorganic and organic arsenic by *Alkaliphilus oremlandii* sp. nov. strain OhILAs. Ann NY Acad Sci.

[bib20] Flemming HC, Wingender J (2010). The biofilm matrix. Nat Rev Microbiol.

[bib21] Gantner S, Andersson AF, Alonso-Sáez L (2011). Novel primers for 16S rRNA-based archaeal community analyses in environmental samples. J Microbiol Meth.

[bib22] Gloag ES, Turnbull L, Huang A (2013). Self-organization of bacterial biofilms is facilitated by extracellular DNA. P Natl Acad Sci USA.

[bib23] Gorlenko V, Tsapin A, Namsaraev Z (2004). *Anaerobranca californiensis* sp. nov., an anaerobic, alkalithermophilic, fermentative bacterium isolated from a hot spring on Mono Lake. Int J Syst Evol Micr.

[bib24] Grant WD, Holtom GJ, O'kelly N (2002). Microbial Degradation of Cellulose-Derived Complexants Under Repository Conditions.

[bib25] Greenfield BF, Moreton AD, Spindler MW (1992). The effects of the degradation of organic materials in the near field of a radioactive waste repository. *Mat Res Symp Proc*.

[bib26] Humphreys P, Laws A, Dawson J (2010). A Review of Cellulose Degradation and the Fate of Degradation Products Under Repository Conditions.

[bib27] Humphreys PN, West JM, Metcalfe R (2010). Microbial Effects on Repository Performance.

[bib28] Jahn A, Nielsen PH (1998). Cell biomass and exopolymer composition in sewer biofilms. Water Sci Technol.

[bib29] Jones BE, Grant WD, Collins NC, Priest FG, Ramos-Cormenzana A, Tindall BJ (1994). Alkaliphiles: diversity and identification. Bacterial Diversity and Systematics.

[bib30] Jung J, Chun J, Park W (2012). Genome sequence of extracellular-protease-producing *Alishewanella jeotgali* isolated from traditional Korean fermented seafood. J Bacteriol.

[bib31] Kim M-S, Jo SK, Roh SW (2010). *Alishewanella agri* sp. nov., isolated from landfill soil. Int J Syst Evol Micr.

[bib32] Kim MS, Roh SW, Nam YD (2009). *Alishewanella jeotgali* sp. nov., isolated from traditional fermented food, and emended description of the genus Alishewanella. Int J Syst Evol Micr.

[bib33] Knill CJ, Kennedy JF (2003). Degradation of cellulose under alkaline conditions. Carbohyd Polym.

[bib34] Kolekar YM, Konde PD, Markad VL (2013). Effective bioremoval and detoxification of textile dye mixture by *Alishewanella* sp. KMK6. Appl Microbiol Biot.

[bib35] Kotelnikova S, Macario AJ, Pedersen K (1998). *Methanobacterium subterraneum* sp. nov., a new alkaliphilic, eurythermic and halotolerant methanogen isolated from deep granitic groundwater. Int J Syst Bacteriol.

[bib36] Libert M, Bildstein O, Esnault L (2011). Molecular hydrogen: an abundant energy source for bacterial activity in nuclear waste repositories. Phys Chem Earth, Parts A.

[bib38] Mcdonald JE, Houghton JN, Rooks DJ (2012). The microbial ecology of anaerobic cellulose degradation in municipal waste landfill sites: evidence of a role for fibrobacters. Environ Microbiol.

[bib37] Maestrojuan GM, Boone JE, Mah RA (1992). Taxonomy and halotolerance of mesophilic *Methanosarcina* strains, assignment of strains to species, and synonymy of *Methanosarcina mazei* and *Methanosarcina frisia*. Int J Syst Bacteriol.

[bib39] Motellier S, Richet C, Merel P (1998). Analysis of cellulose degradation products by capillary electrophoresis. J Chromatogr A.

[bib40] N.D.A. (2010a). Geological Disposal: An Introduction to the Generic Disposal System Safety Case.

[bib41] N.D.A. (2010b). Near-field Evolution Status Report. NDA/RWMD/033.

[bib42] N.D.A. (2013). Radioactive Wastes in the UK: A Summary of the 2013 Inventory.

[bib43] Ordoñez OF, Flores MR, Dib JR (2009). Extremophile culture collection from Andean lakes: extreme pristine environments that host a wide diversity of microorganisms with tolerance to UV radiation. Microbial Ecol.

[bib44] Pikuta EV, Itoh T, Krader P (2006). *Anaerovirgula multivorans* gen. nov., sp. nov., a novel spore-forming, alkaliphilic anaerobe isolated from Owens Lake, California, USA. Int J Syst Evol Micr.

[bib45] Randall M, Rigby B, Thomson O (2013). Assessment of the effects of cellulose degradation products on the behaviour of europium and thorium. Report prepared on behalf of NDA by National Nuclear Laboratory NNL (12) 12239.

[bib46] Rittmann BE, Mccarty PL (2001). Environmental Biotechnology.

[bib47] Rizoulis A, Steele H, Morris K (2012). The potential impact of anaerobic microbial metabolism during the geological disposal of intermediate-level waste. Mineralogical Magazine.

[bib48] Rodrigues DF, Goris J, Vishnivetskaya T (2006). Characterization of *Exiguobacterium* isolates from the Siberian permafrost. Description of *Exiguobacterium sibiricum* sp. nov. Extremophiles.

[bib49] Rout SP, Charles CJ, Garratt EJ (2015). Evidence of the generation of isosaccharinic acids and their subsequent degradation by local microbial consortia within hyper-alkaline contaminated soils, with relevance to intermediate level radioactive waste disposal. PloS One.

[bib50] Rout SP, Radford J, Laws AP (2014). Biodegradation of the alkaline cellulose degradation products generated during radioactive waste disposal. PloS One.

[bib51] Schloss PD, Westcott SL, Ryabin T (2009). Introducing mothur: open-source, platform-independent, community-supported software for describing and comparing microbial communities. Appl Environ Microb.

[bib52] Shaw PB, Robinson GF, Rice CR (2012). A robust method for the synthesis and isolation of β-gluco-isosaccharinic acid ((2R,4S)-2,4,5-trihydroxy-2-(hydroxymethyl)pentanoic acid) from cellulose and measurement of its aqueous pKa. Carbohyd Res.

[bib53] Sridhar J, Eiteman MA (1999). Influence of redox potential on product distribution in Clostridium thermosuccinogenes. Appl Biochem Biotech.

[bib54] Stuart SL, Woods SL, Lemmon TL (1999). The effect of redox potential changes on reductive dechlorination of pentachlorophenol and the degradation of acetate by a mixed, methanogenic culture. Biotechnol Bioeng.

[bib55] Summons RE, Schubotz F, Hays LE (2015). Stable isotope labeling confirms mixotrophic nature of streamer biofilm communities at alkaline hot springs. Front Microbiol.

[bib56] Takai K, Moser DP, Onstott TC (2001). *Alkaliphilus transvaalensis* gen. nov., sp. nov., an extremely alkaliphilic bacterium isolated from a deep South African gold mine. Int J Syst Evol Micr.

[bib57] Tarhriz V, Nematzadeh G, Vahed SZ (2012). *Alishewanella tabrizica* sp. nov., isolated from Qurugöl Lake. Int J Syst Evol Micr.

[bib58] Urbieta MS, González-Toril E, Bazán ÁA (2015). Comparison of the microbial communities of hot springs waters and the microbial biofilms in the acidic geothermal area of Copahue (Neuquén, Argentina). Extremophiles.

[bib59] Van Loon LR, Glaus MA (1997). Review of the kinetics of alkaline degradation of cellulose in view of its relevance for safety assessment of radioactive waste repositories. J Environ Polym Degr.

[bib60] Warwick P, Evans N, Hall T (2003). Complexation of Ni(II) by α-isosaccharinic acid and gluconic acid from pH 7 to pH 13. Radiochim Acta.

[bib61] Williamson AJ, Morris K, Shaw S (2013). Microbial reduction of Fe(III) under alkaline conditions relevant to geological disposal. Appl Environ Microb.

[bib62] Worakit S, Boone DR, Mah RA (1986). *Methanobacterium alcaliphilum* sp. nov., an H_2_-utilizing methanogen that grows at high pH values. Int J Syst Bacteriol.

[bib63] Wu W-M, Jain MK, De Macario EC (1992). Microbial composition and characterization of prevalent methanogens and acetogens isolated from syntrophic methanogenic granules. Appl Microbiol Biot.

