# Supplementary material for: The enrichment of an alkaliphilic biofilm consortia capable of the anaerobic degradation of isosaccharinic acid from cellulosic materials incubated within an anthropogenic, hyperalkaline environment
Source: FEMS Microbiol Ecol. 2015 Jul 20;91(8):fiv085. doi: 10.1093/femsec/fiv085 (PMC4629871; doi:10.1093/femsec/fiv085)
Supplement: Supplementary data are available at FEMSEC online [file femsec_fiv085_index.html]

SUPPLEMENTARY DATA | FEMS Microbiology Ecology

## SUPPLEMENTARY DATA

- SUPPLEMENTARY DATA
