# Supplementary material for: The enrichment of an alkaliphilic biofilm consortia capable of the anaerobic degradation of isosaccharinic acid from cellulosic materials incubated within an anthropogenic, hyperalkaline environment
Source: FEMS Microbiol Ecol. 2015 Jul 20;91(8):fiv085. doi: 10.1093/femsec/fiv085 (PMC4629871; doi:10.1093/femsec/fiv085)
Supplement: Supplementary data are available at FEMSEC online [file supplemental.docx]

**Isolation of an alkaliphilic biofilm consortia cultivated from cellulose within a hyperalkaline environment capable of the anaerobic degradation of isosaccharinic acid**

C.J. Charles^a^, S.P. Rout^a^, E.J. Garrat^a^, K. Patel^a^, A.P. Laws^a^, P,N, Humphreys^a*^.

^a^School of Applied Sciences, University of Huddersfield, Queensgate, Huddersfield HD1 3DH, United Kingdom

* Corresponding author. Tel: +441484472771.

E-mail address: p.n.humphreys@hud.ac.uk (P.N. Humphreys).

## Supplemental information

| Element | Weight% | Atomic% |  |
| --- | --- | --- | --- |
|  |  |  |  |
| C | 26.51 | 40.57 |  |
| O | 41.86 | 48.10 |  |
| Al | 1.26 | 0.86 |  |
| Si | 1.79 | 1.17 |  |
| Ca | 18.14 | 8.32 |  |
| Au | 10.45 | 0.98 |  |
|  |  |  |  |
| Totals | 100.00 |  |  |

Figure S1: EDX analysis of the mineral precipitate formed on the colonised cotton fibre.

Figure S2: Chemistry of pH 11 chloramphenicol amended CDP driven controls.

| Cotton Eubacterial clones | | | |
| --- | --- | --- | --- |
| Clone | Accession number | Closest matching organisms | Sequence similarity (%) |
| ct1 | KP263977 | Clostridium formicaceticum strain DSM 92 | 95 |
| ct2 | KP263978 | Bacillus pseudofirmus OF4 strain OF4 | 99 |
| ct3 | KP263979 | Alkaliphilus oremlandii strain OhILAs | 92 |
| ct4 | KP263980 | Alkaliphilus oremlandii strain OhILAs | 93 |
| ct5 | KP263981 | Bacillus pseudofirmus OF4 strain OF4 | 98 |
| ct6 | KP263982 | Proteiniphilum acetatigenes strain TB107 | 95 |
| ct7 | KP263983 | Clostridium formicaceticum strain DSM 92 | 96 |
| ct8 | KP263984 | Anaerovirgula multivorans strain SCA | 98 |
| ct9 | KP263985 | Anaerovirgula multivorans strain SCA | 97 |
| ct10 | KP263986 | Tissierella praeacuta strain ATCC 25539 | 93 |
| ct11 | KP263987 | Clostridium formicaceticum strain DSM 92 | 95 |
| ct12 | KP263988 | Clostridium formicaceticum strain DSM 92 | 95 |
| ct13 | KP263989 | Erysipelothrix inopinata strain 143-02 16S | 90 |
| ct14 | KP263990 | Clostridium formicaceticum strain DSM 92 | 93 |
| ct15 | KP263991 | Clostridium formicaceticum strain DSM 92 | 94 |
| ct16 | KP263992 | Alkaliphilus oremlandii strain OhILAs | 93 |
| ct17 | KP263993 | Alkaliphilus oremlandii strain OhILAs | 93 |
| ct18 | KP263994 | Anaerovirgula multivorans strain SCA | 98 |
| ct19 | KP263995 | Clostridium formicaceticum strain DSM 92 | 94 |
| ct20 | KP263996 | Anaerovirgula multivorans strain SCA | 94 |
| ct21 | KP263997 | Clostridium formicaceticum strain DSM 92 | 94 |
| ct22 | KP263998 | Tissierella creatinini strain DSM 9508 16S | 93 |
| ct23 | KP263999 | Alishewanella jeotgali strain MS1 | 99 |
| ct24 | KP264000 | Clostridium formicaceticum strain DSM 92 | 94 |
| ct25 | KP264001 | Natronincola peptidivorans strain Z-7031 | 91 |
| ct26 | KP264002 | Anaerovirgula multivorans strain SCA | 95 |
| ct27 | KP264003 | Anaerovirgula multivorans strain SCA | 99 |
| ct28 | KP264004 | Alkaliphilus oremlandii strain OhILAs | 92 |
| ct29 | KP264005 | Clostridium formicaceticum strain DSM 92 | 94 |
| ct30 | KP264006 | Bacillus cohnii strain DSM 6307 | 93 |
| ct31 | KP264007 | Clostridium formicaceticum strain DSM 92 | 91 |
| ct32 | KP264008 | Clostridium formicaceticum strain DSM 92 | 94 |
| ct33 | KP264009 | Alishewanella jeotgali strain MS1 | 96 |
| ct34 | KP264010 | Anaerovirgula multivorans strain SCA | 97 |
| ct35 | KP264011 | Alkaliphilus oremlandii strain OhILAs | 91 |
| ct36 | KP264012 | Clostridium formicaceticum strain DSM 92 | 94 |
| ct37 | KP264013 | Anaerovirgula multivorans strain SCA | 95 |
| ct38 | KP264014 | Anaerovirgula multivorans strain SCA | 95 |
| ct39 | KP264015 | Alkaliphilus oremlandii strain OhILAs | 93 |
| ct40 | KP264016 | Anaerobranca gottschalkii strain LBS3 | 90 |
| ct41 | KP264017 | Tissierella creatinini strain DSM 9508 | 95 |
| ct42 | KP264018 | Dongia mobilis strain LM22 | 88 |
| ct43 | KP264019 | Alkaliphilus oremlandii strain OhILAs | 92 |
| ct44 | KP264020 | Anaerobranca horikoshii strain DSM 9786 | 90 |
| ct45 | KP264021 | Anaerovirgula multivorans strain SCA | 96 |
| ct46 | KP264022 | Ochrobactrum pecoris strain 08RB2639 | 97 |
| ct47 | KP264023 | Bacillus pseudofirmus OF4 strain OF4 | 89 |
| ct48 | KP264024 | Ochrobactrum anthropi strain ATCC 49188 | 99 |
| ct49 | KP264025 | Ochrobactrum anthropi strain ATCC 49188 | 98 |
| ct50 | KP264026 | Corynebacterium marinum strain D7015 | 98 |
| ct51 | KP264027 | Bacillus pseudofirmus OF4 strain OF4 | 91 |
| ct52 | KP264028 | Ochrobactrum anthropi strain ATCC 49188 | 99 |
| ct53 | KP264029 | Pseudochrobactrum asaccharolyticum strain CCUG 46016 | 98 |
| ct54 | KP264030 | Bacillus pseudofirmus OF4 strain OF4 | 99 |
| ct55 | KP264031 | Ochrobactrum anthropi strain ATCC 49188 | 99 |
| ct56 | KP264032 | Alkaliphilus transvaalensis strain SAGM1 | 98 |
| ct57 | KP264033 | Ochrobactrum anthropi strain ATCC 49188 | 97 |
| ct58 | KP264034 | Dietzia cercidiphylli strain YIM 65002 | 99 |
| ct59 | KP264035 | Ochrobactrum anthropi strain ATCC 49188 | 98 |
| ct60 | KP264036 | Gordonia sp. strain KTR9 | 95 |
| ct61 | KP264037 | Corynebacterium marinum strain D7015 | 99 |
| ct62 | KP264038 | Corynebacterium halotolerans strain YIM 70093 | 97 |
| ct63 | KP264039 | Ochrobactrum anthropi strain ATCC 49188 | 99 |
| ct64 | KP264040 | Corynebacterium marinum strain D7015 | 98 |
| ct65 | KP264041 | Corynebacterium marinum strain D7015 | 98 |
| ct66 | KP264042 | Ochrobactrum anthropi strain ATCC 49188 | 96 |
| ct67 | KP264043 | Corynebacterium marinum strain D7015 | 99 |

Table S1: Closest matching organisms and sequence similarities to the Eubacterial colonised cotton clone library.

| Cotton Archaeal clones | | | |
| --- | --- | --- | --- |
| Clone | Accession number | Closest matching organisms | Sequence similarity (%) |
| ct68 | KP264044 | Methanobacterium alcaliphilum strain NBRC 105226 | 99 |
| ct69 | KP264045 | Methanobacterium alcaliphilum strain NBRC 105226 | 99 |
| ct70 | KP264046 | Methanobacterium alcaliphilum strain NBRC 105226 | 99 |
| ct71 | KP264047 | Methanosarcina mazei Go1 | 99 |
| ct72 | KP264048 | Methanobacterium alcaliphilum strain NBRC 105226 | 99 |
| ct73 | KP264049 | Methanobacterium alcaliphilum strain NBRC 105226 | 99 |
| ct74 | KP264050 | Methanobacterium alcaliphilum strain NBRC 105226 | 99 |
| ct75 | KP264051 | Methanobacterium alcaliphilum strain NBRC 105226 | 99 |
| ct76 | KP264052 | Methanomassiliicoccus luminyensis strain B10 | 83 |
| ct77 | KP264053 | Methanobacterium alcaliphilum strain NBRC 105226 | 99 |
| ct78 | KP264054 | Methanobacterium alcaliphilum strain NBRC 105226 | 99 |
| ct79 | KP264055 | Methanobacterium alcaliphilum strain NBRC 105226 | 99 |
| ct80 | KP264056 | Methanomassiliicoccus luminyensis strain B10 | 89 |
| ct81 | KP264057 | Methanobacterium alcaliphilum strain NBRC 105226 | 99 |
| ct82 | KP264058 | Methanobacterium alcaliphilum strain NBRC 105226 | 99 |
| ct83 | KP264059 | Methanobacterium alcaliphilum strain NBRC 105226 | 99 |
| ct84 | KP264060 | Methanobacterium alcaliphilum strain NBRC 105226 | 99 |
| ct85 | KP264061 | Methanobacterium alcaliphilum strain NBRC 105226 | 99 |
| ct86 | KP264062 | Methanomassiliicoccus luminyensis strain B10 | 89 |
| ct87 | KP264063 | Methanobacterium alcaliphilum strain NBRC 105226 | 99 |
| ct88 | KP264064 | Methanobacterium alcaliphilum strain NBRC 105226 | 99 |
| ct89 | KP264065 | Methanobacterium alcaliphilum strain NBRC 105226 | 99 |
| ct90 | KP264066 | Methanobacterium alcaliphilum strain NBRC 105226 | 99 |
| ct91 | KP264067 | Methanobacterium alcaliphilum strain NBRC 105226 | 99 |
| ct92 | KP264068 | Methanomassiliicoccus luminyensis strain B10 | 89 |
| ct93 | KP264069 | Methanobacterium alcaliphilum strain NBRC 105226 | 99 |
| ct94 | KP264070 | Methanobacterium alcaliphilum strain NBRC 105226 | 99 |
| ct95 | KP264071 | Methanobacterium alcaliphilum strain NBRC 105226 | 99 |
| ct96 | KP264072 | Methanobacterium alcaliphilum strain NBRC 105226 | 99 |
| ct97 | KP264073 | Methanobacterium alcaliphilum strain NBRC 105226 | 99 |
| ct98 | KP264074 | Methanobacterium alcaliphilum strain NBRC 105226 | 99 |
| ct99 | KP264075 | Methanobacterium alcaliphilum strain NBRC 105226 | 99 |
| ct100 | KP264076 | Methanobacterium alcaliphilum strain NBRC 105226 | 99 |
| ct101 | KP264077 | Methanobacterium alcaliphilum strain NBRC 105226 | 99 |
| ct102 | KP264078 | Methanobacterium alcaliphilum strain NBRC 105226 | 99 |
| ct103 | KP264079 | Methanobacterium alcaliphilum strain NBRC 105226 | 99 |
| ct104 | KP264080 | Methanobacterium alcaliphilum strain NBRC 105226 | 99 |
| ct105 | KP264081 | Methanobacterium alcaliphilum strain NBRC 105226 | 99 |
| ct106 | KP264082 | Methanobacterium alcaliphilum strain NBRC 105226 | 99 |
| ct107 | KP264083 | Methanobacterium alcaliphilum strain NBRC 105226 | 99 |
| ct108 | KP264084 | Methanobacterium alcaliphilum strain NBRC 105226 | 99 |
| ct109 | KP264085 | Methanobacterium alcaliphilum strain NBRC 105226 | 99 |
| ct110 | KP264086 | Methanobacterium alcaliphilum strain NBRC 105226 | 99 |
| ct111 | KP264087 | Methanobacterium alcaliphilum strain NBRC 105226 | 99 |
| ct112 | KP264088 | Methanobacterium alcaliphilum strain NBRC 105226 | 99 |
| ct113 | KP264089 | Methanobacterium alcaliphilum strain NBRC 105226 | 99 |
| ct114 | KP264090 | Methanobacterium alcaliphilum strain NBRC 105226 | 99 |
| ct115 | KP264091 | Methanobacterium alcaliphilum strain NBRC 105226 | 99 |
| ct116 | KP264092 | Methanobacterium alcaliphilum strain NBRC 105226 | 99 |
| ct117 | KP264093 | Methanobacterium alcaliphilum strain NBRC 105226 | 99 |
| ct118 | KP264094 | Methanobacterium alcaliphilum strain NBRC 105226 | 99 |
| ct119 | KP264095 | Methanobacterium alcaliphilum strain NBRC 105226 | 99 |
| ct120 | KP264096 | Methanobacterium alcaliphilum strain NBRC 105226 | 99 |
| ct121 | KP264097 | Methanobacterium alcaliphilum strain NBRC 105226 | 99 |
| ct122 | KP264098 | Methanobacterium alcaliphilum strain NBRC 105226 | 99 |
| ct123 | KP264099 | Methanobacterium alcaliphilum strain NBRC 105226 | 99 |
| ct124 | KP264100 | Methanobacterium alcaliphilum strain NBRC 105226 | 99 |
| ct125 | KP264101 | Methanobacterium alcaliphilum strain NBRC 105226 | 99 |
| ct126 | KP264102 | Methanobacterium alcaliphilum strain NBRC 105226 | 99 |
| ct127 | KP264103 | Methanobacterium alcaliphilum strain NBRC 105226 | 99 |
| ct128 | KP264104 | Methanobacterium alcaliphilum strain NBRC 105226 | 99 |
| ct129 | KP264105 | Methanobacterium alcaliphilum strain NBRC 105226 | 99 |
| ct130 | KP264106 | Methanobacterium alcaliphilum strain NBRC 105226 | 99 |
| ct131 | KP264107 | Methanobacterium alcaliphilum strain NBRC 105226 | 99 |
| ct132 | KP264108 | Methanobacterium alcaliphilum strain NBRC 105226 | 99 |
| ct133 | KP264109 | Methanobacterium alcaliphilum strain NBRC 105226 | 99 |
| ct134 | KP264110 | Methanobacterium alcaliphilum strain NBRC 105226 | 99 |
| ct135 | KP264111 | Methanobacterium alcaliphilum strain NBRC 105226 | 99 |

Table S2: Closest matching organisms and sequence similarities to the Archaeal colonised cotton clone library.

| pH 11 microcosm Eubacterial clones | | | |
| --- | --- | --- | --- |
| Clone | Accession number | Closest matching organisms | Sequence similarity (%) |
| A01 | KP728118 | Alishewanella jeotgali strain MS1 | 99 |
| A02 | KP728119 | Alishewanella jeotgali strain MS1 | 96 |
| A03 | KP728120 | Alishewanella jeotgali strain MS1 | 99 |
| A05 | KP728121 | Alishewanella jeotgali strain MS1 | 95 |
| A06 | KP728122 | Alishewanella jeotgali strain MS1 | 99 |
| A07 | KP728123 | Alishewanella jeotgali strain MS1 | 98 |
| A08 | KP728124 | Alishewanella jeotgali strain MS1 | 99 |
| A09 | KP728125 | Alishewanella jeotgali strain MS1 | 97 |
| A11 | KP728126 | Alishewanella jeotgali strain MS1 | 98 |
| A12 | KP728127 | Alishewanella jeotgali strain MS1 | 98 |
| B01 | KP728128 | Alishewanella jeotgali strain MS1 | 99 |
| B02 | KP728129 | Alishewanella jeotgali strain MS1 | 98 |
| B03 | KP728130 | Alishewanella jeotgali strain MS1 | 98 |
| B04 | KP728131 | Alishewanella jeotgali strain MS1 | 99 |
| B09 | KP728132 | Alishewanella jeotgali strain MS1 | 96 |
| B12 | KP728133 | Alishewanella jeotgali strain MS1 | 97 |
| C01 | KP728134 | Alishewanella jeotgali strain MS1 | 98 |
| C05 | KP728135 | Alishewanella jeotgali strain MS1 | 98 |
| C07 | KP728136 | Alishewanella jeotgali strain MS1 | 99 |
| C08 | KP728137 | Alishewanella jeotgali strain MS1 | 99 |
| C09 | KP728138 | Alishewanella jeotgali strain MS1 | 99 |
| C12 | KP728139 | Alishewanella jeotgali strain MS1 | 98 |
| D01 | KP728140 | Alishewanella jeotgali strain MS1 | 99 |
| D03 | KP728141 | Alishewanella jeotgali strain MS1 | 98 |
| D05 | KP728142 | Alishewanella jeotgali strain MS1 | 98 |
| D06 | KP728143 | Alishewanella jeotgali strain MS1 | 99 |
| D07 | KP728144 | Alishewanella jeotgali strain MS1 | 98 |
| D08 | KP728145 | Alishewanella jeotgali strain MS1 | 97 |
| D09 | KP728146 | Alishewanella jeotgali strain MS1 | 99 |
| D11 | KP728147 | Alishewanella jeotgali strain MS1 | 98 |
| E01 | KP728148 | Alishewanella jeotgali strain MS1 | 97 |
| E02 | KP728149 | Alishewanella jeotgali strain MS1 | 96 |
| E04 | KP728150 | Bacillus pseudofirmus OF4 strain OF4 | 98 |
| E06 | KP728151 | Alishewanella jeotgali strain MS1 | 93 |
| E07 | KP728152 | Alishewanella jeotgali strain MS1 | 98 |
| E08 | KP728153 | Alishewanella jeotgali strain MS1 | 95 |
| E12 | KP728154 | Alkaliphilus crotonatoxidans strain B11-2 | 98 |
| F01 | KP728155 | Alishewanella jeotgali strain MS1 | 96 |
| F02 | KP728156 | Alishewanella jeotgali strain MS1 | 97 |
| F03 | KP728157 | Alishewanella jeotgali strain MS1 | 98 |
| F05 | KP728158 | Alishewanella jeotgali strain MS1 | 97 |
| F06 | KP728159 | Alishewanella jeotgali strain MS1 | 99 |
| F07 | KP728160 | Alishewanella jeotgali strain MS1 | 98 |
| F09 | KP728161 | Alishewanella jeotgali strain MS1 | 99 |
| F10 | KP728162 | Alishewanella jeotgali strain MS1 | 95 |
| F12 | KP728163 | Alishewanella jeotgali strain MS1 | 99 |
| G01 | KP728164 | Alishewanella jeotgali strain MS1 | 96 |
| G05 | KP728165 | Alishewanella jeotgali strain MS1 | 98 |
| G06 | KP728166 | Alishewanella jeotgali strain MS1 | 97 |
| G07 | KP728167 | Bacillus pseudofirmus OF4 strain OF4 | 99 |
| G08 | KP728168 | Alishewanella jeotgali strain MS1 | 97 |
| G09 | KP728169 | Alishewanella jeotgali strain MS1 | 98 |
| G10 | KP728170 | Alishewanella jeotgali strain MS1 | 98 |
| G11 | KP728171 | Alishewanella jeotgali strain MS1 | 99 |
| H01 | KP728172 | Alishewanella jeotgali strain MS1 | 96 |
| H04 | KP728173 | Alishewanella jeotgali strain MS1 | 95 |
| H05 | KP728174 | Alishewanella jeotgali strain MS1 | 98 |
| H10 | KP728175 | Alishewanella jeotgali strain MS1 | 98 |
| H12 | KP728176 | Alishewanella jeotgali strain MS1 | 99 |

Table S3: Closest matching organisms and sequence similarities to the Eubacterial pH 11 microcosm clone library.

| Day | CFU/mL | SE | Biomass (mg DW) | SE | Biomass (mg C) | SE |
| --- | --- | --- | --- | --- | --- | --- |
| 0 | 9.50E+06 | 1.23E+06 | 1.69E+00 | 2.18E-01 | 8.32E-01 | 1.08E-01 |
| 7 | 1.35E+07 | 3.18E+05 | 2.40E+00 | 5.64E-02 | 1.18E+00 | 2.78E-02 |
| 14 | 1.46E+07 | 6.61E+05 | 2.59E+00 | 1.17E-01 | 1.28E+00 | 5.79E-02 |

Table S4: Results from ATP quantification from pH 11 microcosm at different time points during the sampling period
